# Pancreaticoduodenectomy for an Ampullary Region Carcinoma Occurred in Annular Pancreas Coexistent with Replaced Common Hepatic Artery

**DOI:** 10.1155/2018/3258141

**Published:** 2018-02-20

**Authors:** Ryuji Komine, Atsushi Shimizu, Kazuhiko Mori, Keisuke Minamimura, Toru Hirata, Takashi Kobayashi, Nobuo Toda, Masaya Mori

**Affiliations:** ^1^Department of General and Gastrointestinal Surgery, Mitsui Memorial Hospital, 1 Kanda Izumicho, Chiyoda-Ku, Tokyo 101-8643, Japan; ^2^Department of Gastroenterology, Mitsui Memorial Hospital, 1 Kanda Izumicho, Chiyoda-ku, Tokyo 101-8643, Japan; ^3^Department of Pathology, Mitsui Memorial Hospital, 1 Kanda Izumicho, Chiyoda-ku, Tokyo 101-8643, Japan

## Abstract

**Introduction:**

Annular pancreas is a rare congenital abnormality characterized by a ring of pancreatic tissue surrounding the descending portion of the duodenum. Annular pancreas coexisting with replaced common hepatic artery which is also a rare anatomical variation has not been reported previously.

**Case Presentation:**

A 53-year-old man visited our hospital complaining of epigastric pain. Based on radiological examinations, he was diagnosed as having pancreatitis, annular pancreas, and hepatomesenteric trunk. One month later, obstructive jaundice developed. Endoscopic examination revealed ampullary region carcinoma. We performed pancreaticoduodenectomy using the “artery-first” approach.

**Discussion:**

Both annular pancreas and common hepatic artery anomaly are rare. High-quality preoperative imaging and awareness of such rare conditions are necessary for operative safety. Although the embryological relationship between these anomalies is uncertain, the present case may suggest some relevance between the two.

**Conclusion:**

The “artery-first” approach may be a useful method for pancreaticoduodenectomy in patients who have an anatomical abnormality.

## 1. Introduction

Annular pancreas (AP) is a rare congenital anomaly in which the second part of the duodenum is surrounded by a ring of pancreatic tissue continuous with the head of the pancreas. The prevalence of AP was reported in 3 of 20,000 autopsies and 3 of 24,519 surgical cases [1, 2]. Although pancreatitis, duodenal obstruction, and so on are known as symptoms of AP, approximately two-thirds of the patients are asymptomatic [3]. AP is also regarded as one of the risk factors of pancreatobiliary malignancy. However, replaced common hepatic artery (CHA) arising from the superior mesenteric artery (SMA) was reportedly seen in 1.13% of 19,013 cases [4]. The hepatomesenteric trunk is also rarely encountered but can be problematic in pancreaticoduodenectomy (PD). Although the relationship between these rare anomalies is uncertain, embryologically, they are both considered to occur during the same week of gestation [5]. We herein describe an extremely rare case in which these anomalies coexisted in a patient with ampullary region carcinoma treated by PD with the “artery-first” approach.

## 2. Case Presentation

**Figure 1:**
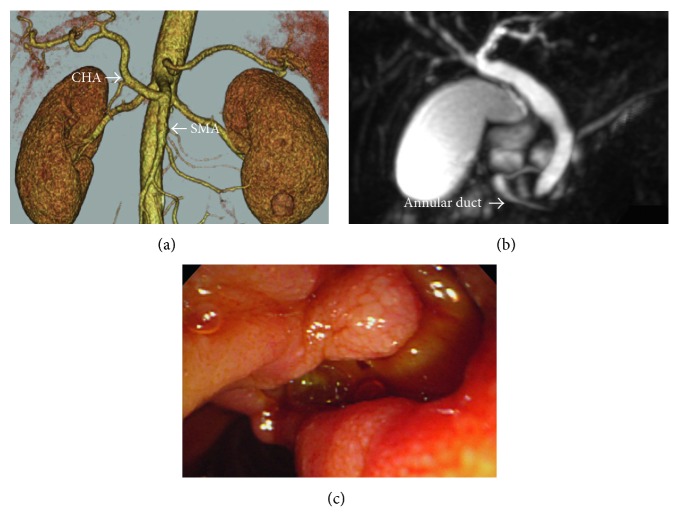
Preoperative image findings. (a) Three-dimensional computed tomography scan of the abdomen reveals the replaced common hepatic artery arising from the superior mesenteric artery. (b) Magnetic resonance cholangiopancreatogram shows the annular duct, but no relevant tumor or dilation of the common bile duct and the pancreatic duct. (c) Esophagogastroduodenoscopy shows an ulcer at the ampulla of Vater, and the biopsy results of the ulcer indicate adenocarcinoma. Cannulation of the papilla region could not be performed because of direct invasion of the tumor.

A 53-year-old man presented with epigastric pain visited our hospital. Result of the blood analysis indicated hyperamylasemia. The abdominal computed tomography (CT) scan showed diffuse parenchymal enlargement, and he was diagnosed as having mild pancreatitis according to the modified CT severity index. The CT scan also revealed an anomaly of the CHA arising from the SMA (Figure 1(a)) and annular pancreatic duct. Magnetic resonance cholangiopancreatography (MRCP) confirmed that the annular duct formed a loop in the head of the pancreas (Figure 1(b)). There were no pancreaticobiliary maljunction. Neither the common bile duct nor the main pancreatic duct was dilated. Endoscopic ultrasonography was performed to investigate other causes, but the ampulla of Vater could not be observed due to technical difficulty.

One month later, result of the follow-up laboratory test demonstrated obstructive jaundice. The CT scan revealed a dilatation of the common bile duct. An ulcerative tumor was found around the duodenal papilla region by upper esophagogastroduodenoscopy (Figure 1(c)). Biopsy results from the ulcer indicated adenocarcinoma. Endoscopic retrograde cholangiography (ERCP) was performed to investigate the biliary or pancreatic duct, but cannulation of the ampulla of Vater could not be performed successfully due to the invasion of the tumor.

**Figure 2:**
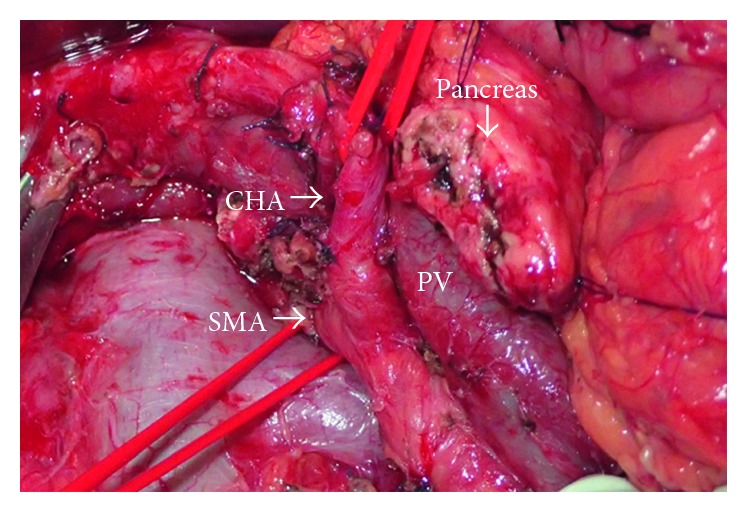
Intraoperative findings. The common hepatic artery is arising from the superior mesenteric artery and runs posterior to the pancreas.

Subtotal stomach-preserving pancreaticoduodenectomy with dissection of the regional lymph nodes was performed. Laparotomy showed that the pancreatic parenchymal tissue surrounded the descending part of the duodenum. Neither liver metastasis nor peritoneal dissemination was found. The tumor was located in the head of the pancreas. The SMA was exposed first to identify the origin of the CHA (the “artery-first” approach). The CHA arouse from posteriorly to pancreatic head and traveled posteriorly to the portal vein (PV), and then it ran anteriorly to the PV after it branched off the gastroduodenal artery and anterior superior pancreaticoduodenal artery (Figure 2). The pancreas was transected at the neck directly overlying the PV-SMV-splenic vein confluence.

**Figure 3:**
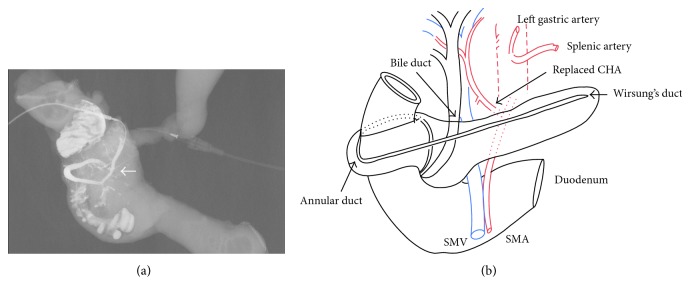
Findings of the resected specimen. (a) Cholangiopancreatogram showing cannulation of the resected specimen and injection of urografin from the stump of the main pancreatic duct with the annular duct encircling the descending part of the duodenum and draining at the major papilla (←). (b) Schematic illustration of the pancreatic duct and replaced common hepatic artery, based on the ventral and dorsal pancreas.

**Figure 4:**
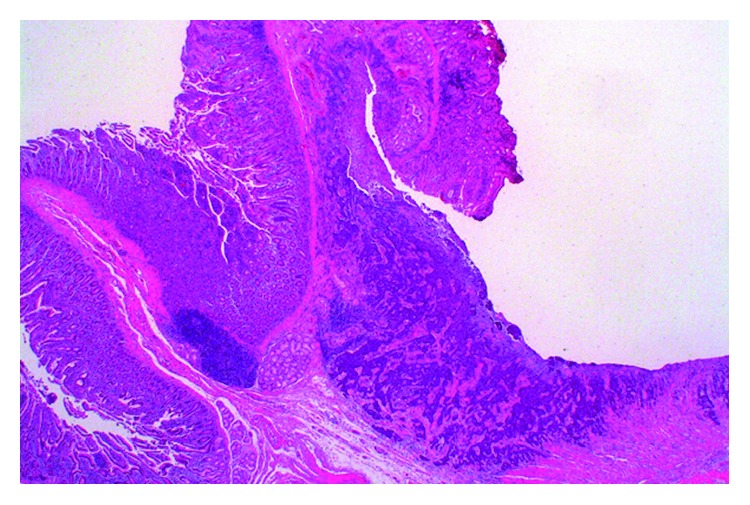
Microscopic findings. Tumor-forming ulcer at the papilla region, propagating with cord-like and alveolar structures. It consisted of poorly differentiated adenocarcinoma with the formation of a partially glandular structure.

Cholangiopancreatography of the resected specimen showed that the annular duct was connected to the major papilla (Figures 3(a) and 3(b)). The final pathology revealed poorly differentiated adenocarcinoma (Figure 4) without regional lymph node metastasis (International Union Against Cancer TNM classification, 8th edition: T3N0M0, IIA). The patient's postoperative course was uneventful, and he was discharged 21 days postoperatively. He is doing well without any evidence of recurrence at the most recent follow-up of 6 months postoperatively.

## 3. Discussion

Recently, with modern diagnostic imaging devices, such as CT, MRCP, or ERCP, 1 in 250 examined cases was found to have AP [6, 7]. About 700 cases of AP have been reported in the literatures [8], and a relationship with the increasing incidence of pancreatobiliary malignancy also has been described [8–11]. Among the cases reported in the English literatures, there are eight resected cases of ampullary region carcinoma associated with AP (Table 1) [11–17]. It should be important to raise the suspicion of potential malignancy when symptoms occur in adult patients who have AP and have been asymptomatic.

Yogi et al. classified the annular pancreas as six variants of the ductal anatomy. In accordance with this classification, type 2, in which the duct of Wirsung encircles the duodenum but drains at the major papilla, corresponds with our case [18]. ERCP is usually useful to diagnose pancreatic duct; however, in our case, the transpapillary approach or cannulation to the pancreatic duct could not be performed because of direct invasion of the carcinoma. MRCP may be another diagnostic option for AP, but it cannot always identify the minor papilla or thin Santorini duct. A contrast study of the resected specimen was the most informative. In our case, AP did not become a technical problem during the operation because the pancreas was transected at the distal side of the AP. It is also important to clearly identify the dilated main pancreas pancreatic duct to understand the path of the annular duct before the operation for safe resection.

Michels described the hepatic arterial anatomy and variations of 200 cadaveric dissections and identified 10 types of hepatic arterial anatomy [19]. Our case was classified as type IX. Accidental ligation of the hepatic arteries can lead to hepatic dysfunction, ischemia of bilioenteric anastomosis, and may result in a fatal leak. From a technical perspective, the “artery-first” approach was also useful in the case of anomaly. Sanjay et al. described types and advantages of six “artery-first” approaches [20]. We used the posterior approach; that is, we exposed and taped the root of the SMA initially in front of the left renal vein after kocherization. This enabled total control of the arterial anomaly before identification and ligation, which contributed to safe resection and secure lymph node dissection even in an extremely rare condition.

Our case may suggest some embryological relevancies between AP and hepatic arterial anomalies. The developmental mechanism of AP has not been determined, but Lecco's theory, in which the adherence of the ventral bud to the duodenal wall prior to rotation is regarded as the causes, seems to be more accepted than other theories [21]. The hepatomesenteric trunk is considered to build due to degeneration and adhesion of ventral splanchnic arteries [22]. Both anatomical anomalies are formed during the same fifth to seventh week of gestation. Fukai et al. reported that all of the pancreatic tissue of the AP may be derived from the ventral anlage, and presented a hypothesis about Yogi's type 2 AP based on Lecco's theory. According to the theory, incomplete rotation, overgrowth of the tip of the ventral anlage, and adhesion to the dorsal anlage could make the annular duct fused with the main duct of the dorsal anlage instead of crossing over the lower bile duct [23]. In addition, the CHA anomaly was reported to sometimes pass through the pancreatic head and running in front of the main pancreatic duct in those cases [19, 24, 25]. In our case, the CHA passed behind the head of the pancreas, on the left side of the annular duct. It may be rational to consider that the rotation and adhesion of the pancreatic bud occur somewhat later than the formation of the vascular structure, and the vasculature is one of the regulators that determine the position of the fusion of the main pancreatic duct and annular duct.

## Figures and Tables

**Table 1:** Summary of reported cases of ampullary region carcinoma associated with AP.

Case	Author	Age	Sex	Symptoms	Operation	TNM classification	Size (cm)
1	Transveldt et al. [12]	80	F	Jaundice, WL	PD	ND	ND
2	Benger and Thompson [13]	66	M	Jaundice, WL	PD	T2N0M0	ND
3	Rathnaraj et al. [11]	55	M	Jaundice	PD	ND	2 × 2
4	Shan et al. [14]	40	F	Jaundice, AP	PD	ND	ND
5	Shan et al. [14]	45	M	Jaundice, AP	PPPD	ND	ND
6	Foo et al. [15]	78	F	Jaundice, AP	PD	T1N0M0	1.2 × 3.5
7	Yazawa et al. [16]	59	M	ED	PPPD	TisN0M0	2.5 × 2.0
8	Tewari et al. [17]	42	F	Jaundice, AP	PD	ND	2.0 × 2.0
9	Present case	53	M	Epigastric pain	SSPPD	T2N0M0	3.3 × 3.0

PPPD: pylorus-preserving pancreaticoduodenectomy; SSPPD: subtotal stomach-preserving pancreaticoduodenectomy; WL: weight loss; AP: abdominal pain; ED: epigastric discomfort; ND: not described.

## Abbreviations

AP: Annular pancreas
CHA: Common hepatic artery
SMA: Superior mesenteric artery
PD: Pancreaticoduodenectomy
CT: Computed tomography
MRCP: Magnetic resonance cholangiopancreatography
EGD: Esophagogastroduodenoscopy
ERCP: Endoscopic retrograde cholangiography.
